# Association of lower serum irisin levels with diabetes mellitus: Irrespective of coronary collateral circulation, and syntax score

**DOI:** 10.14744/nci.2021.73669

**Published:** 2021-12-07

**Authors:** Aydin Akyuz, Beysim Mert, Demet Ozkaramanli Gur, Muhammet Mucip Efe, Huseyin Aykac, Seref Alpsoy, Savas Guzel

**Affiliations:** 1. Department of Cardiology, Namik Kemal University Faculty of Medicine, Tekirdag, Turkey; 2. Department of Cardiovascular Physiology, Namik Kemal University Institute of Health Sciences, Tekirdag, Turkey; 3. Department of Biochemistry, Namik Kemal University Faculty of Medicine, Tekirdag, Turkey

**Keywords:** Coronary circulation, diabetes mellitus, irisin

## Abstract

**Objective:**

Irisin is a myokine thought to be involved in the pathophysiological process of atherosclerosis with its’ cardiovascular protective effects. Patients with diabetes mellitus (DM) have lower levels of irisin. Therefore, we investigated whether there is a connection between irisin, DM, coronary collateral circulation (CCC), and SYNTAX scores representing coronary artery disease (CAD) severity.

**Methods:**

This study evaluated 86 patients who have at least one epicardial coronary artery with chronic total occlusion. We included Rentrop 0–1 into the poor CCC group (n=45) and Rentrop 2–3 into the good CCC group (n=41) and measured serum irisin levels.

**Results:**

Irisin levels did not differ (17585 [882–37741] pg/ml and (17504 [813–47683] pg/ml, p=0.772) between the two groups. Irisin levels were lower in patients with diabetes (n=41; 14485 [813–29398] pg/ml) than those without diabetes (n=45; 19724 [865–47683] pg/ml (p=0.002). Irisin was not correlated with SYNTAX scores. In multivariate analysis, DM (OR=0.463; CI: 0.184–0.783; p=0.012) was a negative predictor of good CCC development

**Conclusion:**

Although its level is decreased in patients with diabetes, serum irisin levels have no role in the pathophysiology of collateral development and CAD severity.

**W**ell-developed coronary collateral circulation (CCC) is considered to be a natural by-pass vessel that reduces the ischemic area of the myocardium and that increases life expectancy [1]. Even if there is complete stenosis among patients with the same coronary artery stenosis, the degree of CCC is variable; therefore, the development of CCC lacks a definitive mechanism. Many studies have been carried out in this field with the discovery of new biomarkers. Growth factors, angiogenetic regulators, inflammatory cytokines, endothelial chemokines, adhesion molecules, and oxidative stress are shown to affect new vessel development [2–7]. The well-established clinical predictors of good CCC were severe coronary artery stenosis [5], absence of diabetes mellitus (DM) [6], and male gender [7]. Induction of angiogenesis and re-vascular development has importance in predicting future cardiovascular events. CCC is evaluated subjectively using the Cohen-Rentrop method on coronary angiography [8].

Irisin, firstly described by Boström et al. [9], is a myokine that has effects on the cardiovascular system and that has been extensively studied in recent years as a new biomarker of atherosclerosis. Myokines are often released from skeletal muscles and have autocrine, paracrine, and endocrine effects [10]. Irisin acts as an insulin-sensitizing hormone at the cell level and consists of a fibronectin Type III domain-containing protein 5(FNDC5) that is a plasma membrane protein.

In recent years, the studies showing the relationship between serum irisin level and coronary artery disease (CAD) have been conducted [11, 12]. In these studies, serum levels of reduced irisin were correlated with increased coronary artery severity, and irisin levels are low in patients with DM [13–15]. However, the relationship between serum irisin levels, CCC, and lesion complexity has not been studied so far. Considering the association of DM with lower irisin levels and the patients with DM who have usually poor CCC, we, therefore, decided to investigate whether this relationship exists.

## Materials and Methods

### Selection of Cases

**Figure 1.**
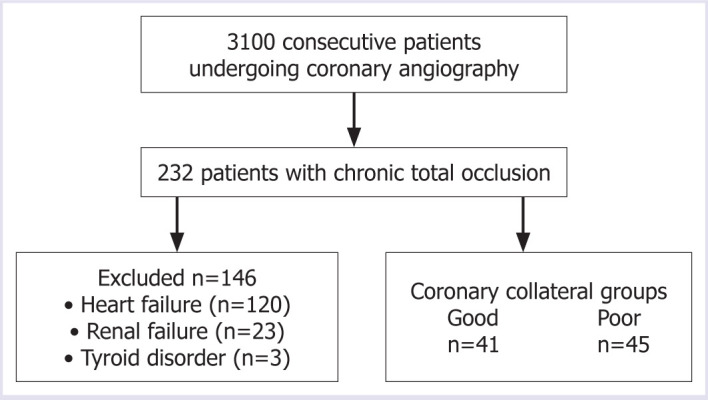
The flow chart in the study.

This cross-sectional study was conducted between March 2018 and August 2019. Namik Kemal University Faculty of Medicine Ethics Committee was obtained before the study (Ethics Committee Decision No: 2018.18.02.03). All participants included in the study were informed about the study, and an informed consent was obtained. The study’s flowchart was presented in Figure 1. All patients underwent coronary angiography due to symptoms and findings consistent with ischemia in resting electrocardiography, echocardiography, or exercise stress test. Participants were selected by three cardiologists who were blinded to the study protocol. Our exclusion criteria were those under the age of 18 and older than 75, patients who had no total occlusion in at least one epicardial coronary artery, those with rhythm disturbances, intracardiac conduction defect or branch block, left ventricular systolic dysfunction with left ventricle ejection fraction (LVEF%) ≤35%, chronic renal failure (estimated glomerular filtration rate <60 ml/min/1.73 m^2^), acute or chronic inflammation, chronic lung or liver disease, and thyroid disorder or cancer disease.

**Highlight key points**

• Irisin, a glycoprotein, acts as an insulin-sensitizing hormone which has a key role in energy production and tissue healing
• Irisin was not associated with the number of involved coronary artery lesions
• DM, especially in those with medication on insulin, associates with a lower irisin level that is not related to CCC and lesion complexity.

Current medication with beta-blockers, acetylsalicylic acid, statin, calcium-channel blockers, and angiotensin-converting enzyme inhibitors, and angiotensin receptor blockers were recorded. Coronary angiograms were evaluated according to Rentrop collateral classification [16]. Those with class 2 (n=26) and 3 (n=15) were included in the good CCC group and those with class 0 (n=11) and 1 (n=34) were enrolled to poor CCC group. Demographic characteristics such as age, weight, height, body mass index (BMI), gender, waist circumference, history of smoking, presence of DM, hypertension, and hyperlipidemia were also recorded.

DM was diagnosed as fasting blood glucose above 126 mg/dL or use of any antidiabetic medication. The diagnosis of hypertension was accepted as systolic pressure ≥140 mmHg and diastolic pressure ≥90 mmHg or antihypertensive drug use. Diagnosis of hyperlipidemia was established as total cholesterol (TC) ≥200 mg/dl, low-density lipoprotein cholesterol (LDL-C) ≥130 mg/dl, triglyceride ≥150 mg/dl, high-density lipoprotein cholesterol (HDL-C) ≤40 mg/dl. LVEF% was measured by the modified Simpson method using transthoracic echocardiography. Left ventricle mass index (LVMI) was calculated using linear method, and was indexed to body surface area [17].

### Coronary Angiographic Examination

Coronary angiography procedures were performed using the standard protocol in our cardiac catheterization laboratory. After femoral artery puncture using the Seldinger method left, right six, and seven F Judkins catheters were used. For the left coronary artery at least four positions (Spider position, right oblique, anterior-posterior cranial, and left oblique) and the right coronary artery at least two positions (Left oblique and left cranial) were taken in at least 80 images at a rate of 25 frames per second. Additional exposures were obtained for suspicious lesions to confirm the degree of severity. 6–8 ml of opaque material was given by hand for each exposure. For this purpose, 50–100 cc nonionic radiopaque material was used for each patient. Coronary anatomical examination recordings were taken by coronary angiography device (Artiz Zee, Siemens company, Munich, Germany).

We defined chronic total occlusion (CTO) as the absence of flow in the epicardial coronary artery that has been completely occluded for more than 3 months. Of all CTO patients had prior coronary angiography performed more than 3 months ago confirming the presence of CTO.

The SYNTAX score is the most recent scoring system to assess coronary artery severity and lesion complexity. The online SYNTAX score calculator was used (http://syntaxscore.com) which was shown to have good reproducibility [18]. For intraobserver variability, 10 randomly selected patients were re-evaluated by the same observer blinded to the first measurements; for inter-observer variability, these patients were reevaluated by a second observer. The inter-and intra-observer correlation coefficients of SYNTAX were ≥0.95. We assessed coronary collateralization by Rentrop score instead of collateral flow, an invasive, and better index. However, the Rentrop score is a reliable and reproducible method to evaluate coronary collateral vessels. Rentrop CCC classification is represented as follows [8, 16]: grade 0, no visible collateral vessels filling; grade 1, minimal visible side branches of collateral vessels; grade 2, collateral vessels fill the epicardial artery partially; and grade 3, collateral vessels fill the epicardial artery. Grades 0 and 1 were enrolled in the poor CCC group, while Grades 2 and 3 were enrolled in the good CCC group.

### Blood Collection and Biochemical Analysis

Fasting blood samples were taken from participants before coronary angiography. Routine blood biochemistry was carried out as part of the standard procedure. The patients were asked not to exercise except for short-distance walking within 1 h before blood samples were taken. Samples were spared for irisin examination. Glucose, LDL-C, HDL-C, TC, triglyceride, creatinine, uric acid, high-sensitivity C reactive protein (CRP), and hemogram measurements were performed in routine biochemical tests. Since the irisin could deteriorate while waiting for the blood sample, the serum sample was stored after 2 h to be stored at -80 °C, and then each patients’ irisin levels were analyzed after centrifugation at 4000 rpm for 15 min.

Serum irisin levels were measured using the commercially available enzyme-linked immunosorbent assay (ELISA) kit – (Irisin ELISA kit, Bioassay Technology Laboratory Ltd, Shanghai, China). According to the information obtained in the catalog of the factory producing kits, the lowest detectable irisin coefficient variability is given as below 8% (product catalog no: E-3253Hu and detection range: 0.2–60 ng/ml).

### Statistical Analysis

Variable data were analyzed by SPSS 18 software (SPSS Inc, Chicago, Illinois, USA). Shapiro –Wilk distribution test was used to evaluate whether the variable distributions were parametric or not. For the normal distribution, the student test was used for comparison of the two groups and non-normally distributed variables were compared with the Mann–Whitney U test. A Chi-square test was used for categorical variables. If the expected number of categorical variables in any group was <5, the Fischer test p-value was accepted. The correlations of the irisin levels (nonparametric variable) with other variables were determined by the Spearman correlation test. Kruskal–Wallis test was used to compare irisin levels according to Rentrop classification. Univariate and multivariate logistic regression analyses were performed to detect the predictors of good CCC. Linear regression analyzes were performed to determine the variables that could affect the irisin level. A p<0.05 was considered significant.

## Results

When the characteristics of good (n=41) and poor (n=45) CCC groups were compared; age, sex ratio, BMI, waist circumference, diabetes, hyperlipidemia, hypertension, smoking, and family history rates were similar (All p>0.05). Serum values of biochemical variables such as glucose, TC, TG, LDL-C, HDL-C, creatinine, and serum CRP levels of groups did not differ (p>0.05 for all values) (Table 1). Serum irisin levels were similar between good collateral and poor collateral groups (17585 [882–37741] pg/ml and (17504 [813–47683] pg/ml, p=0.772). The rates of multi-vessel disease (p=0.045) were higher in the good CCC group (Table 2). Serum irisin levels were negatively correlated with triglyceride levels (r=–0.282; p=0.009) and fasting glucose (r=–0.209; p=0.046). Irisin was not correlated with age, BMI, TC, LDL-C, HDL-C, creatinine, CRP, LVEF, LVMI, and SYNTAX score (p>0.05 for all) (Table 3).

**Table 1.** Comparison of demographic, anthropological, and biochemical properties of the good and poor collateral groups

Variables	Good collateral group n=41	Poor collateral group n=45	p
Age, year	62.3±5.6	59±5.3	0.104*
Female/Male	8/33	6/39	0.512^†^
BMI, kg/m^2^	27.8±5.1	27.7±4.3	0.982*
Waist circumference, cm	82.2±8.2	83.7±7.6	0.756*
DM, %	46.3	48.9	0.813^†^
Hypertension, %	51.2	18 (40	0.297^†^
Hyperlipidemia, %	68.3	66.7	0.872^†^
Smoking, %	68.3	73.3	0.607^†^
Family history of CAD	73.2	66.7	0.512^†^
Fasting glucose, mg/dl	119 (69–418)	130 (79–347)	0.243^‡^
TC, mg/dl	174 (72–481)	169 (86–289)	0.470^‡^
Triglycerides, mg/dl	196±117	198±109	0.910*
HDL-cholesterol, mg/dl	42 (29–83)	43 (23–80)	0.483^‡^
LDL-cholesterol, mg/dl	87.5 (43–347)	92837–162)	0.551^‡^
Creatinine, mg/dl	0.84 (0.61–1.32)	0.91 (0.7–1.29)	0.218^‡^
Serum irisin, pg/ml	17585 (882–37741)	17504 (813–47683)	0.772^‡^
Hs-CRP, mg/dl	5.6 (0.2–9.3)	4.9 (0.1–9.8)	0.905^‡^
LVEF %	58.5±5.2	56.8±4.4	0.266*
LV mass index, g/m^2^	88.6±21.7	86.2±16.7	0.730*
Drug use, %			
Beta blockers	46.3	55.6	0.393^†^
Calcium channel blockers	43.9	16	0.429^†^
Acetyl salicylic acid	87.8	86.7	0.875^†^
Statins	70.7	73.3	0.788^†^
ACEI/ARB	51.2	44.4	0.530^†^
Oral antidiabetics	19.5	28.9	0.312^†^
Insulin	12.2	11.1	0.876^||^

*: Student t-test; †: Chi-square test; ‡: Mann-Whitney U test; ||: Fisher’s test; ACEI/ARB: Angiotensin-converting-enzyme inhibitor/Angiotensin-receptor blocker; CAD: Coronary artery disease; Hs-CRP: High sensitive C reactive protein; LVEF: Left ventricle ejection fraction; HDL: High-density lipoprotein; LDL: Low-density lipoprotein; LVMI: Left ventricle mass index; TC: Total cholesterol; DM: Diabetes mellitus; BMI: Body mass index.

**Table 2.** Coronary angiography characteristics of the study population

Variables	Good collateral group (n=41) %	Poor collateral group (n=45) %	p
1 vessel disease	24.4	44.5	0.204
2 vessel disease	41.5	40	0.890
3 vessel disease	34.1	15.6	0.045
SYNTAX score	21 (5–40.5)	3–34	0.156
Rentrop collateral grades			
0		24.5	
1		75.6	
2	63.4		
3	36.6		

*: Chi-square test.

**Table 3.** Correlation of irisin values with clinical parameters

Variables	Correlation coefficient (r*)	p
Age	0.062	0.571
BMI	-0.011	0.954
Waist circumference	-0.023	0.864
Fasting glucose	-0.209	0.046
TC		-0.101	0.353
Triglycerides	-0.282	0.009
HDL-cholesterol	0.148	0.175
LDL-cholesterol	-0.081	0.461
Creatinine	-0.014	0.902
High sensitive CRP	-0.035	0.753
LVEF	-0.024	0.876
LVMI	-0.083	0.446
SYNTAX score	0.119	0.294

*: Spearman correlation test; CRP: C reactive protein; LVEF: Left ventricle ejection fraction; LVMI: Left ventricle mass index; TC: Total cholesterol; BMI: Body mass index; HDL: High-density lipoprotein; LDL: Low-density lipoprotein.

**Figure 2.**
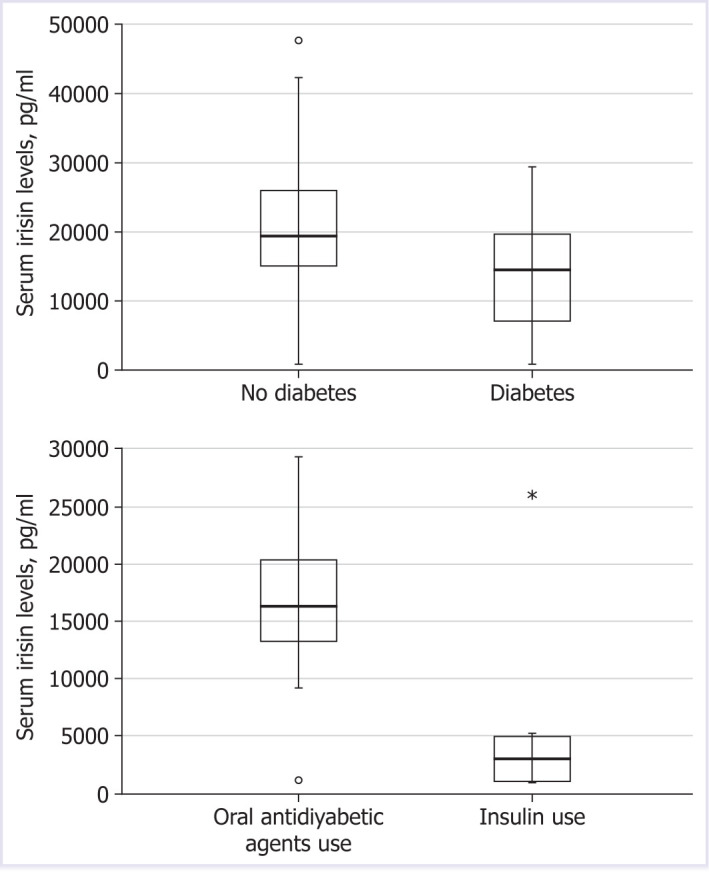
Serum irisin levels according to diabetes mellitus and non-diabetics, or medication in patients with coronary artery disease.

**Figure 3.**
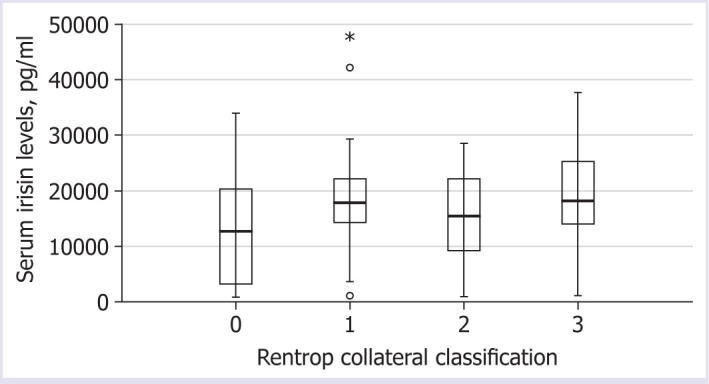
Comparison of irisin levels according to Rentrop groups.

Comparison of irisin values in the study population according to demographic and angiographic characteristics showed that its’ levels of the patients with hypertension, hyperlipidemia, smoking, family history, and multi-vessel disease were shown to be similar compared to that of the ones without these characteristics. However, patients with diabetes (n=41) (14485 [813–29398] pg/ml) had lower irisin levels than those without diabetes (n=45; 19724 [865–47683] pg/ml) (p=0.002).The subgroup analysis in patients with diabetes has further revealed that those who use oral antidiabetic agents (n=23) had higher irisin levels compared to those on insulin therapy (n=18) (16975 [1089–29398] vs. 4880 [813–26078], pg/ml, p<0.001) (Table 4 and Fig. 2). Irisin levels were similar among Rentrop groups, (Rentrop 0: 13.556±11.904, Rentrop 1:9.192±1.600, Rentrop 2:15.272±8.444, and Rentrop 3:20186±10155, pg/ml respectively, p=0.176) (Fig. 3).

**Table 4.** Comparison of irisin values in the study population according to demographic and angiographic characteristics

Variables	n	Irisin levels* Median (Min–Max)
Female	14	17432 (813–34793)
Male	72	23631 (917–47683)
P	0.235	
DM+	45	19724 (865–47683)
DM-	41	14485 (813–29398)
p	0.002	
Insulin	18	4880 (813–26078)
Oral antidiabetic agents	23	16975 (1089–29398)
p	<0.001	
Hypertension +	39	15926 (813–34793)
Hypertension -	47	19192 (865–47683)
p	0.195	
Hyperlipidemia +	58	17432 (813–42277)
Hyperlipidemia −	28	19048 (2597–47683)
p	0.381	
Smoking +	61	17361 (813–67200)
Smoking −	25	19505 (917–67200)
p	0.898	
Family history of CAD +	60	15596 (813–37741)
Family history of CAD -	26	18727 (882–47683)
p	0.102	
Multivessel disease >2+	56	17076 (813–47683)
Multivessel disease >2-	30	17361 (865–34037)
p	0.768	

*: Mann-Whitney U test; Min: Minimum; Max: Maximum; DM: Diabetes mellitus; CAD: Coronary artery disease.

In multivariate analysis for good CCC development, the presence of DM (OR=0.463; CI: 0.184–0.783; p=0.012) was a negative predictor in the study (Table 5).

**Table 5.** Univariate and multivariate logistic regression analyses for the presence of good CCC

Univariate analyses	p	OR	95% CI
Female gender	0.555	0.699	0.213–2.296
DM	0.012	0.398	0.143–0.583
Hypertension	0.345	1.541	0.601–3.987
Hyperlipidemia	0.809	1.121	0.444–2.827
Smoking	0.669	1.231	0.475–3.192
Family history of CAD	0.304	1.667	0.630–4.412
Presence of CTO	0.010	3.529	2.362–5.612
Multivessel disease>2	0.101	2.231	0.855–5.823
Irisin levels	0.768	0.992	0.896–1.103
Multivariate analyses			
DM	0.012	0.463	0.184–0.783

DM: Diabetes mellitus; CAD: Coronary artery disease; CCC: Coronary collateral circulation; CI: Confidence interval; CTO: Chronic total occlusion; OR: Odds ratio.

## Discussion

The most important finding of our study was that serum irisin levels were found to be significantly lower in diabetic patients and lowest in patients with insulin therapy. The presence of DM was a negative variable of good CCC development, which is in parallel with previous studies [5, 6].

There was no relationship between irisin, CCC, and coronary artery lesion complexity determined by the SYNTAX score, which was the main question of our study. Irisin was not associated with the number of involved coronary artery lesions. Efe et al. [12] found an association between irisin levels and SYNTAX scores. We, however, have not shown such an association between irisin and SYNTAX scores. In addition to the severity of epicardial CAD, endothelial dysfunction and microvascular atherosclerosis are also important determinants of the inflow of blood to the myocardium [19]. Furthermore, some patients with visually normal coronary angiograms have various degrees of atherosclerotic plaque [20]. Although the levels of this myokine are theoretically expected to be compatible with the epicardial blood flow to the myocardium, other factors that determine the microvascular flow and the response of the irisin cell receptors need to be considered. SYNTAX score alone; therefore, is not the single determinant of myocardial ischemia. These reasons can partly explain why we could not show a correlation between SYNTAX score and irisin. The main purpose of our study was to determine the relationship between irisin, collateral circulation, and SYNTAX score; therefore, we neglected the relationship between irisin and ischemia. Moreover, Efe et al. [12]. have excluded diabetic patients, in whom microvascular atherosclerosis is more prominent, from the study which could further explain the discrepancy of our results.

In a meta-analysis, Guo et al. [21] have concluded that irisin levels are decreased in CAD patients. There is a meta-analysis in the literature showing that irisin levels are low in CAD patients, and namely, there are fewer CAD lesions at high irisin levels, which suggests that irisin is cardioprotective [22–24]. In light of these previous studies, even though irisin has cardioprotective effects, the association of its with CCC and SYNTAX scores in patients with CAD may not be shown.

Consistent with previous studies, our study, found irisin levels to be low in diabetics. The relationship between BMI and insulin resistance is well-known. We found that serum irisin levels were inversely correlated with glucose and triglyceride levels, but not with BMI. Conflicting publications are claiming that irisin increases and decreases in subjects with obesity [15, 23]. However, our findings showed that there was no relationship between BMI and irisin levels. In an earlier study, serum irisin levels were not correlated with CRP and inversely correlated with glucose and triglycerides [25]. Our study confirmed these previous findings.

Serum irisin levels increase with exercise [26]. It is also known that irisin accelerates wound healing [22] and cell-mediated cardiac repair and reduces apoptosis as a cardiac progenitor [23]. Irisin is considered a cardioprotective glycoprotein hormone [24]. However, irisin has no antihypertensive effects on hypertensive rats [27]. Unfortunately, diabetics cannot obtain benefits from irisin-induced cardioprotection. There are still no animal and human experiments that can clearly show why irisin is decreased in diabetic patients. Irisin may have therapeutic potential in diabetic patients. Irisin is released from skeletal and heart muscle, adipose tissue, liver, kidney, nerve sheaths, skin, and subcutaneous tissue [28, 29]. It triggers the conversion of dense white fat cells under the skin to brown fat cells. The greater number of brown fat cells makes weight loss possible. White fat cells which are dense store energy in patients with diabetes and obesity, while brown fat cells break down for energy and are more sensitive to insulin. We, surprisingly, found the lowest irisin levels in patients with diabetes who use insulin. The patients who are in the stage of requiring insulin are considered to possess more severe illness. In patients with DM using insulin, the adverse effects on the synthesis of irisin at the cell level due to DM may be more than in non-insulin users. There is a need for further studies to reveal the cause of decreased irisin levels in diabetics and those on insulin medication.

A decrease in circulating irisin levels leads to a decrease in energy levels and further induce ischemia in the heart tissue [9, 10]. Irisin runs the pump called the uncoupling protein-1 in the mitochondria of the white fat cells and increases mitochondrial ATP production and increases heat production and provides glucose balance. In other words, it accelerates the production and consumption of energy [14, 29]. Because irisin is released from intact myocytes and other organs to protect the energy stores of the ischemic myocardium, we can theoretically consider that irisin levels are decreased in patients with DM who have CAD due to its inadequate release to meet the need for further ATP production.

### Study Limitations

This study has some limitations. The main limitation of the study is the small number of the study population. Irisin is also secreted from skeletal muscle; however, we did not calculate the skeletal muscle index and the daily exercise characteristics of the patients. Having a control group with normal coronary arteries would allow us to compare irisin levels in subjects with and without CAD. The investigation of the correlation between serum irisin level and long-term mortality could have also contributed to the validation of our findings.

### Conclusion

Irisin is a glycoprotein that has cardioprotective properties. Although irisin is low in diabetic patients, it is not related to CCC and lesion complexity. The exact cause of its’ decreased levels in diabetic patients with medication on insulin is unclear.
